# Vaginal washing and lubrication among female sex workers in the Mexico-US border region: implications for the development of vaginal PrEP for HIV prevention

**DOI:** 10.1186/s12889-018-5946-z

**Published:** 2018-08-14

**Authors:** Heather A. Pines, Shirley J. Semple, Steffanie A. Strathdee, Craig W. Hendrix, Alicia Harvey-Vera, Pamina M. Gorbach, Carlos Magis-Rodríguez, Gustavo Martinez, Thomas L. Patterson

**Affiliations:** 1 Department of Medicine, University of California, San Diego, 9500 Gilman Drive, MC 0507, La Jolla, CA 92093 USA; 2 Department of Psychiatry, University of California, San Diego, 9500 Gilman Drive, MC 0680, La Jolla, CA 92093 USA; 3 Department of Medicine, Johns Hopkins University, 600 N. Wolfe Street, Baltimore, MD 21287 USA; 4 Department of Epidemiology, University of California, Los Angeles, 650 Charles E Young Drive S, BOX 951772, Los Angeles, CA 90095 USA; 5 Centro Nacional para la Prevención y Control del VIH/SIDA (CENSIDA) Ministry of Health, Mexico City, Mexico; 6 Federacion Mexicana de Asociaciones Privadas, Ciudad Juarez, Chihuahua, Mexico

**Keywords:** Vaginal practices, Pre-exposure prophylaxis, HIV, Female sex workers, Mexico

## Abstract

**Background:**

To assess the potential acceptability and inform the development of behaviorally-congruent vaginal douche- or gel-based HIV pre-exposure prophylaxis (PrEP) products, we examined vaginal washing and lubrication practices among female sex workers (FSWs) in Tijuana and Ciudad Juarez, two northern Mexico cities bordering the United States (US).

**Methods:**

Two hundred and ninety-five HIV-negative FSWs (145 Tijuana; 150 Ciudad Juarez) enrolled in a behavioral HIV prevention intervention trial completed surveys assessing vaginal washing and lubrication practices, as well as motivators and barriers to performing each practice. Logistic regression was used to identify potential predictors of each practice in the past month.

**Results:**

In the past month, vaginal washing was performed by 56 and 22% of FSWs in Tijuana and Ciudad Juarez (*p* <  0.0001), respectively, while vaginal lubrication was performed by 64 and 45% of FSWs in Tijuana and Ciudad Juarez (*p* = 0.001), respectively. Vaginal washing was positively associated with living in Tijuana (adjusted odds ratio [AOR] = 4.35, 95% confidence interval [CI]: 2.60–7.30), older age (AOR = 1.04 per year, 95% CI: 1.01–1.06), and vaginal lubrication (AOR = 2.99, 95% CI: 1.67–5.35), while it was negatively associated with being born in the same state as the study site (AOR = 0.50, 95% CI: 0.31–0.82), earning a monthly income ≥3500 pesos (AOR = 0.53, 95% CI: 0.28–1.00), and hazardous alcohol consumption (AOR = 0.56, 95% CI: 0.33–0.95). Vaginal lubrication was positively associated with living in Tijuana (AOR = 2.21, 95% CI: 1.37–3.54) and vaginal washing (AOR = 2.91, 95% CI: 1.64–5.18), while it was negatively associated with being born in the same state as the study site (AOR = 0.47, 95% CI: 0.29–0.75).

**Conclusions:**

The moderate and high prevalence of vaginal washing and lubrication, respectively, suggest behaviorally-congruent, multi-purpose, vaginal douche- and gel-based PrEP products that simultaneously address FSWs’ needs and prevent HIV infection may be acceptable to many FSWs along the Mexico-US border. Future product development and implementation should also consider the link between vaginal washing and lubrication to ensure existing practices do not undermine vaginal PrEP product effectiveness.

**Trial registration:**

ClincialTrials.gov (NCT02447484).

**Electronic supplementary material:**

The online version of this article (10.1186/s12889-018-5946-z) contains supplementary material, which is available to authorized users.

## Background

Female sex workers (FSWs) are at substantial risk of HIV infection in low- and middle-income countries (LMICs) where their odds of infection are estimated to be 13.5 times those of reproductive-aged women [1]. FSWs’ vulnerability to HIV infection is often heightened by structural factors that limit their ability to negotiate condom use with male clients, including gender inequality, violence, poverty, social marginalization, and sex work criminalization [2]. As such, user-controlled HIV prevention methods delivered in the context of comprehensive HIV prevention packages that also include behavioral and structural interventions could dramatically reduce HIV incidence among FSWs [3].

Antiretroviral (ARV) pre-exposure prophylaxis (PrEP) products are user-controlled HIV prevention methods that can be formulated as oral pills, long-acting injectables, or topical gels, douches, suppositories/tablets, films, or rings for vaginal or rectal application [4–6]. Oral tenofovir-based PrEP is a highly effective HIV prevention strategy [7–10] and efforts to bring its implementation to scale are underway globally [11]. Topical PrEP products are in various stages of development. PrEP formulated as vaginal dapivirine-containing rings confers moderate protection against HIV infection [12, 13] and is currently under review for market approval by the European Medicines Agency. However, due to inconsistent findings with respect to the efficacy of PrEP formulated as tenofovir-containing gels among women [14–16], the future of topical PrEP remains uncertain. Although the mixed findings reported to-date have primarily been attributed to insufficient levels of adherence in trials unable to demonstrate efficacy [17], recent evidence suggests that the vaginal microbiome may also have played a role [18]. More specifically, lower vaginal concentrations of tenofovir were observed among women with bacterial vaginosis (BV) associated, non-*Lactobacillus* dominant vaginal microbiota [18, 19], which were shown to metabolize tenofovir in in vitro studies [18]. Vaginal concentrations of dapivirine, on the other hand, do not appear to be impacted by the presence of BV-associated bacteria [20]. Taken together, these findings suggest that the efficacy of dapivirine-containing vaginal PrEP products may not be affected by the vaginal microbiome, while adherence to more frequent dosing regimens may be required for tenofovir-containing vaginal PrEP products to confer protection against HIV among women with non-*Lactobacillus* dominant vaginal microbiota [18]. Nevertheless, the body of evidence to-date suggests that regardless of the ARV formulation and its impact on the vaginal microbiome, adherence will be critical to vaginal PrEP’s real-world effectiveness.

PrEP product acceptability or desirability are crucial to adherence. While longer-acting systemic product formulations (e.g., oral, injectable) may be appealing to some potential users, others may prefer short-acting, non-systemic topical products (e.g., gels, douches, suppositories/tablets, films) that can be used on-demand before and after sex. Mack et al. [21] have argued that just as the introduction of more contraceptive methods provided women with more options to choose from and ultimately increased their use of contraception [22], the development and implementation of multiple PrEP product formulations may similarly enhance PrEP uptake and adherence, and thus maximize its potential impact on HIV incidence at the population-level [21]. Therefore, as PrEP product development advances, acceptability research with diverse potential user populations must continue to inform the design of candidate products such that they are specific to potential users’ needs and biology in order to facilitate uptake and adherence among those who could benefit most from their use.

Although FSWs have not been well represented in oral or vaginal PrEP efficacy trials [3], previous research suggests that intravaginal practices, including intravaginal cleansing (cleaning or washing inside the vagina using fingers, cloth, or a douching device) and intravaginal insertion (pushing or placing something inside the vagina, such as lubricants, using fingers, cloth, or an applicator) [23], are common among FSWs globally [24–29]. As such, there may be a role for behaviorally-congruent vaginal PrEP products formulated as gels or douches within this vulnerable population. Intravaginal practices vary across regions and cultures and are influenced by a number of socio-contextual factors [30–32], but are often motivated by hygiene, health, and sexuality [33]. Among FSWs, intravaginal practices are further motivated by a desire to prevent STIs and meet clients’ expectations related to cleanliness and sexual pleasure [34, 35]. Because intravaginal cleansing has been linked to several adverse gynecologic and reproductive health outcomes, BV, HIV, other sexually transmitted infections (STIs) [36–40], previous research has also examined women’s intravaginal practices to inform the development of acceptable vaginal PrEP products that are also safe and effective [23, 32]. However, few studies distinguish between intravaginal practices, and little is known about the practice-specific behaviors associated with FSWs’ existing vaginal practices and whether they could be leveraged to deliver multi-purpose, vaginal gel- or douche-based products that simultaneously address FSWs’ needs and prevent HIV infection.

This study was designed to address this gap in knowledge by examining vaginal washing and vaginal lubrication practices among FSWs in Tijuana and Ciudad Juarez, Mexico. These northern Mexico border cities have established commercial sex work industries and are positioned on major drug trafficking routes into the United States (US), which have contributed to the emergence of dynamic and overlapping epidemics of substance use and HIV infection in the Mexico-US border region [41, 42]. While HIV prevalence among reproductive-aged adults in Mexico is estimated to be 0.2% [43], it has been estimated to be 6% among FSWs in the Mexico-US border region [44] and as high as 12% among those who also inject drugs [45]. High rates of gonorrhea (6%), chlamydia (13%), and syphilis (14%) have also been documented among FSWs in the border region [44]. In a survey conducted with FSWs who inject drugs in Tijuana and Ciudad Juarez between 2008 and 2010, 53% of FSWs in Ciudad Juarez and 33% of FSWs in Tijuana reported performing intravaginal practices in the past 6 months most commonly to clean (65%) or treat vaginal symptoms (64%) [28]. While that work established the prevalence of intravaginal practices among FSWs in the region, practice-specific data on vaginal washing and vaginal lubrication were not collected. As such, the present study builds on that preliminary work to inform the potential development and implementation of safe and effective, behaviorally-congruent vaginal gel- or douche-based PrEP products acceptable to FSWs in the Mexico-US border region.

## Methods

This study was conducted from July 2016 to January 2017 among 313 FSWs in Tijuana (*n* = 163) and Ciudad Juarez (*n* = 150) enrolled in a randomized controlled trial evaluating the efficacy of a text messaging intervention designed to sustain the effect of an interactive, single session sexual risk reduction counseling session demonstrated to be efficacious among FSWs in the Mexico-US border region [46, 47]. Potential participants were recruited at known sex work locations (e.g., bars, brothels, street corners) in each city. Interested individuals were invited to undergo eligibility screening at each city’s study site - an unmarked office building located in Tijuana’s red-light district and a clinical setting located in downtown Ciudad Juarez. Eligibility criteria included: female sex; ≥18 years of age; exchange of money, drugs, or other goods for sex in the past month; condom-unprotected vaginal or anal sex with a male client in the past month; HIV-negative; willing to receive antibiotic treatment if positive for chlamydia, gonorrhea, or syphilis; and cell phone ownership. Because a higher HIV prevalence has been documented among FSWs who inject drugs in the border region [45], FSWs were enrolled such that approximately one-third reported injection drug use, one-third reported non-injection drug use, and one-third did not report any drug use or only reported marijuana use, which will allow the parent study to examine whether drug use modifies the efficacy of the intervention. After providing written informed consent at enrollment, participants underwent HIV/STI testing, completed a baseline survey, participated in the interactive sexual risk reduction counseling session, and were randomized to receive behavior maintenance text messages (intervention) or general health text messages (control). Rapid HIV/STI test results were delivered to participants at the baseline visit, and participants were asked to return within 1 month to receive their confirmatory HIV/STI test results. At that time, participants also completed a supplemental survey to elicit information on their vaginal washing and vaginal lubrication practices. All study procedures were approved by ethics committees at the University of California, San Diego, Xochicalco University in Tijuana, and SADEC-FEMAP in Ciudad Juarez.

### Data collection

Surveys were administered by local, Spanish speaking interviewers using computer-assisted personal interviewing (CAPI). The baseline survey took ~ 50 min to complete and collected information on socio-demographics (age; birthplace; education; average monthly income; marital status; number of children), non-commercial sex partners (spouse or steady partner; casual partners; anonymous partners; frequency of condom-protected and condom-unprotected vaginal sex with those partners in the past month) sex work characteristics (primary sex work location; amount earned for condom-protected and condom-unprotected vaginal sex with clients; history of client perpetrated sexual coercion or physical abuse; number of regular and non-regular clients in the past month; frequency of condom-protected and condom-unprotected vaginal sex with clients in the past month; alcohol and illicit drug use during sex with clients in the past month), hazardous alcohol consumption (Alcohol Use Disorders Identification Test [AUDIT] [48] score ≥ 8, which is consistent with the definition used in previous research with FSWs [49, 50]), past month and lifetime illicit drug use – types of drugs used (marijuana; heroin; inhalants; methamphetamine; ecstasy; cocaine; speedball [heroin + cocaine]; Mexican speedball [heroin + methamphetamine]; tranquilizers; barbiturates; other), frequency of use in the past month (once per month, 2–3 days per month, once per week, 2–3 days per week, 4–6 days per week, everyday), and routes of administration in the past month (ingested, injected, smoked/sniffed, other) [47, 51, 52], and reproductive and sexual health (routine gynecological exam in the past 6 months; contraceptive use other than condoms in the past 6 months; HIV/STI testing in the past 6 months; current vaginal symptoms; current pain during vaginal sex).

The supplemental survey took ~ 35 min to complete at participants’ follow-up visits to receive their HIV/STI test results, which typically occurred within 1 month of baseline. Prior to asking questions about vaginal washing and vaginal lubrication during the supplemental survey, these practices were defined for participants using definitions adapted from those developed by the World Health Organization Gender, Sexuality, and Vaginal Practices Study Group for intravaginal cleansing and intravaginal insertion [23]. Vaginal washing was defined as washing inside the vagina with commercial solutions, soap and water, or other household products (e.g., baking soda, disinfectants) using fingers, cloth, or a douching device to pump the solution inside the vagina. Vaginal lubrication was defined as pushing or placing products, such as creams, oils, or sexual lubricants, inside the vagina using fingers, cloth, or an applicator.

The supplemental survey elicited information about vaginal washing and vaginal lubrication via separate questions specific to each practice. Questions about vaginal washing referred to washing in general as well as before and after vaginal sex, while questions about vaginal lubrication referred only to lubrication before or during vaginal sex. Participants were asked how often they practiced vaginal washing/lubrication, when they practiced vaginal washing (before vaginal sex, after vaginal sex, around menstruation, while bathing), application methods or devices used for vaginal washing/lubrication, and commercial and non-commercial solutions/products used for vaginal washing/lubrication. Pictures were shown to participants to clarify terms used to assess specific application methods. To facilitate recall of the commercial solutions/products used, participants were also shown a list with pictures of 23 commercially available solutions for vaginal washing and 150 commercially available products for vaginal lubrication. The list of commercial solutions/products was compiled after an exhaustive search of solutions/products for sale in local stores as well on the Internet. Participants who reported using vaginal lubricants in the past month were also asked whether they or their clients/sex partners supplied the vaginal lubricants used, whether they or their clients/sex partners applied the vaginal lubricants used, where vaginal lubricants were applied, how often vaginal lubricant application interrupted sex, and the types of vaginal lubricants used (water-based, silicone-based, or oil-based). Finally, participants who reported vaginal washing/lubrication in the past month indicated via 5-point Likert scale responses (strongly disagree, disagree, neither agree nor disagree, agree, strongly agree) the extent to which potential motivators of vaginal washing/lubrication represented reasons they engaged in these practices. Conversely, those who did not report vaginal washing/lubrication in the past month indicated via the same 5-point Likert scale responses the extent to which potential barriers to vaginal washing/lubrication represented reasons they did not engage in these practices. Open-ended questions were also used to elicit other motivators and barriers to performing vaginal washing and lubrication.

### HIV/STI testing

Participants underwent rapid antibody testing for HIV (Advance Quality Anti-HIV 1&2; InTec Products, Inc) and syphilis (Advance Quality Anti-TP; InTec Products, Inc). Rapid test results were delivered to participants within ~ 20 min, and those whose rapid test results were positive underwent confirmatory HIV (Architect HIV Ag/Ab Combo; Abbott; Geenius™ HIV ½ Supplemental Assay; Bio-Rad;) and/or syphilis (rapid plasma reagin (RPR) test: BD Macro-Vue™ RPR; Becton, Dickinson and Company; Treponemal (TP) assay: Architect Syphilis TP assay; Abbott) testing. RPR and TP positive participants with titers ≥1:8 were considered active syphilis cases. Urine samples were collected to detect *Chlamydia trachomatis* and *Neisseria gonorrhoeae* infections via nucleic acid amplification testing (Aptima Combo 2® Assay; Hologic). The San Diego County Public Health Laboratory conducted all nucleic acid amplification and confirmatory testing, the results of which were delivered to participants within approximately 1 month. STI-positive participants were offered free treatment according to Mexican STI treatment guidelines and HIV-positive participants were referred to municipal health clinics in Tijuana and Ciudad Juarez for free care and treatment.

### Statistical analysis

Descriptive statistics (i.e., proportions for categorical variables; medians and interquartile ranges [IQRs] for continuous variables) were calculated by study site to characterize our sample of FSWs, as well as their vaginal washing and lubrication practices and reported motivators and barriers to performing these practices. Logistic regression was used to examine socio-demographics, sex work characteristics, substance use behaviors, and reproductive and sexual health factors as predictors of our outcomes of interest, vaginal washing and vaginal lubrication practices in the past month. Potential predictors significantly associated with vaginal washing or lubrication practices in bivariate analyses (*p*-value< 0.05) were further examined in multivariable models. In previous research, age, education, number of previous pregnancies, number of clients, condom use with clients, HIV testing, and history of STIs have been associated with FSWs’ intravaginal practices [24, 27, 28, 30]. Based on this research, directed acyclic graphs (DAGs) [53] were constructed to depict known or plausible relationships between the potential predictors and the outcomes. These DAGs were then used to identify confounders for inclusion in multivariable models examining the effect of each potential predictor on the outcome (s). Because confounders of the effect of one predictor on an outcome could mediate the effect of another predictor on the outcome, separate multivariable models were constructed to estimate the total effect of each predictor on an outcome [54, 55]. This resulted in five progressive sets of adjustment variables (see Table 5 footnotes). Interaction terms between the predictors of interest and study site were examined, but stratified results are not presented as findings were similar across study sites. All statistical analyses were performed using SAS 9.4 (SAS Institute, Inc.; Cary, NC).

## Results

### Sample characteristics

To ensure the relevance of data collected at baseline to our analysis of past month vaginal washing and vaginal lubrication, we restricted our analysis sample to 295 participants (145 Tijuana; 150 Ciudad Juarez) who completed the supplemental survey within 3 months of their baseline visit (median time between visits: 24.0 days; interquartile range [IQR] = 20.0–34.0). Overall, our sample had a median age of 38.0 years (IQR = 30.0–46.0), with 44% reporting at least a secondary school education and 27% reporting that they were married or in a common-law relationship (Table 1). There were several differences between participants in Tijuana and Ciudad Juarez with respect to socio-demographics, non-commercial sex partners, sex work characteristics, substance use behaviors, and reproductive and sexual health. More participants in Ciudad Juarez reported an average monthly income ≥3500 pesos (96% vs. 43%; *p*-value< 0.0001) and being born in the same state as the study site (76% vs. 25%; *p*-value< 0.0001). A similar proportion of participants in Tijuana (37%) and Ciudad Juarez (27%) reported having a spouse or steady partner (*p*-value = 0.09), while more participants in Tijuana reported non-commercial casual sex partners (44% vs. 18%; *p*-value< 0.0001) and anonymous sex partners (15% vs. 2%; *p*-value< 0.0001). Nearly two-thirds of participants in Tijuana and Ciudad Juarez reported illicit drug use in the past month, with more participants in Tijuana reporting methamphetamine use (55% vs. 8%; *p*-value< 0.0001) and more participants in Ciudad Juarez reporting cocaine use (49% vs. 5%; *p*-value< 0.0001). Eighty-one percent of participants in Tijuana reported being street-based sex workers, while 69% of participants in Ciudad Juarez reported working primarily out of hotels or motels (*p*-value< 0.0001). The median number of clients reported in the past month did not differ significantly between participants in Tijuana (median = 40.0; IQR = 19.0–70.0) and Ciudad Juarez (median = 46.5; IQR = 28.0–76.0) (*p*-value = 0.07). However, Ciudad Juarez participants reported a lower median percent of vaginal sex acts with clients that were condom-protected in the past month (median = 50%; IQR = 20–80% vs. 70%; IQR = 50–90%; *p*-value< 0.0001) and more use of contraceptives other than condoms in the past 6 months (81% vs. 17%; *p*-value< 0.0001). More participants in Tijuana reported HIV/STI testing in the past 6 months (40% vs. 27%; *p*-value = 0.02), current vaginal symptoms (41% vs. 24%; *p*-value = 0.001), and current pain during sex (19% vs. 4%; *p*-value< 0.0001). Twenty-eight percent of participants in Tijuana tested positive for any STI compared to only 10% of participants in Ciudad Juarez (*p*-value< 0.0001), with more participants in Tijuana testing positive for chlamydia (20% vs. 8%), gonorrhea (13% vs. 1%), and active syphilis (6% vs. 1%).

**Table 1:** Characteristics of HIV-negative female sex workers in Tijuana and Ciudad Juarez, Mexico

	Tijuana		Ciudad Juarez		Total		*p*-value
	*N* = 145		*N* = 150		*N* = 295		
	n	%	n	%	n	%	
Sociodemographics							
Median age (years)	38.0	IQR = 31.0–48.0	37.0	IQR = 29.0–44.0	38.0	IQR = 30.0–46.0	0.17
Born in same state as study site	36	24.8	114	76.0	150	50.9	< 0.0001
Completed secondary school	61	42.1	69	46.0	130	44.1	0.50
Married/common-law relationship	36	24.8	43	28.7	79	26.8	0.46
Median number of children	3.0	IQR = 1.0–4.0	3.0	IQR = 2.0–4.0	3.0	IQR = 2.0–4.0	0.62
Average monthly income ≥3500 pesos (~ 172 USD)	62	42.8	144	96.0	206	69.8	< 0.0001
Non-commercial sexual partners							
Spouse or steady partner	53	36.6	41	27.3	94	31.9	0.09
Median % of VS acts with condoms (past month)	0.0	IQR = 0.0–0.0	0.0	IQR = 0.0–0.0	0.0	IQR = 0.0–0.0	0.48
Any casual partners (past month)	63	43.5	27	18.0	90	30.5	< 0.0001
Median % of VS acts with condoms (past month)	0.5	IQR = 0.0–1.0	0	IQR = 0.0–0.0	0.0	IQR = 0.0–1.0	0.0001
Any anonymous partners (past month)	22	15.2	3	2.0	25	8.5	< 0.0001
Median % of VS acts with condoms (past month)	0.5	IQR = 0.0–1.0	0	IQR = 0.0–0.0	0.5	IQR = 0.0–1.0	0.07
Sex work characteristics							
Main sex work location							< 0.0001
Bar or nightclub	27	18.6	37	24.7	64	21.7	
Street	117	80.7	5	3.3	122	41.4	
Hotel or motel	1	0.7	103	68.7	104	35.3	
Other	0	0.0	5	3.3	5	1.7	
Median difference in USD earned for condomless VS	5.0	IQR = 0.0–10.0	3.0	IQR = 0.0–5.0	5.0	IQR = 0.0–7.5	0.51
Ever forced to have sex or physically abused by a client	53	36.6	54	36.0	107	36.3	0.92
Median # of clients (past month)	40.0	IQR = 19.0–70.0	46.5	IQR = 28.0–76.0	44.0	IQR = 23.0–74.0	0.07
Median # regular clients	7.0	IQR = 4.0–15.0	4.0	IQR = 2.0–7.0	5.0	IQR = 3.0–10.0	< 0.0001
Median # non-regular clients	24.0	IQR = 9.0–56.0	40.0	IQR = 21.0–70.0	32.0	IQR = 12.0–63.0	< 0.0001
Median % of VS acts with clients with condoms (past month)	0.7	IQR = 0.5–0.9	0.5	IQR = 0.2–0.8	0.6	IQR = 0.3–0.9	< 0.0001
Median % of VS acts with regular clients with condoms	0.6	IQR = 0.3–0.9	0.0	IQR = 0.0–0.6	0.4	IQR = 0.0–0.8	< 0.0001
Median % of VS acts with non-regular clients with condoms	0.9	IQR = 0.5–1.0	0.6	IQR = 0.3–0.9	0.8	IQR = 0.4–1.0	< 0.0001
Any illicit drug use during sex with a client (past month)	53	36.6	69	46.0	122	41.4	0.10
Any alcohol use during sex with a client (past month)	44	30.3	103	68.7	147	49.8	< 0.0001
Substance Use							
Illicit drug use (past month)	88	60.7	94	62.7	182	61.7	0.73
Methamphetamine	79	54.5	12	8.0	91	30.9	< 0.0001
Cocaine	7	4.8	74	49.3	81	27.5	< 0.0001
Marijuana	42	29.0	34	22.7	76	25.8	0.22
Heroin	37	25.5	27	18.0	64	21.7	0.12
Tranquilizers	19	13.1	29	19.3	48	16.3	0.15
Mexican speedball (methamphetamine + heroin)	20	13.8	0	0.0	20	6.8	< 0.0001
Speedball (cocaine + heroin)	1	0.7	7	4.7	8	2.7	0.07
Inhalants	3	2.1	6	4.0	9	3.1	0.50
Ecstasy	3	2.1	2	1.3	5	1.7	0.68
Barbiturates	0	0.0	0	0.0	0	0.0	–
Injection drug use (past month)	39	26.9	31	20.7	70	23.7	0.21
Hazardous alcohol consumption (past 12 months)	57	39.3	79	52.7	136	46.1	0.02
Reproductive/sexual health							
Routine gynecological exam (past 6 months)	16	11.0	20	13.3	36	12.2	0.55
Contraceptive use other than condoms (past 6 months)	25	17.2	121	80.7	146	49.5	< 0.0001
Tubal ligation	3	12.0	78	64.5	81	55.5	
Birth control pill	8	32.0	16	13.2	24	16.4	
Intrauterine device	10	40.0	9	7.4	19	13.0	
Hormone injection (i.e., Depo-Provera)	2	8.0	10	8.3	12	8.2	
Other	2	8	9	7.4	11	7.5	
HIV/STI testing (past 6 months)	58	40.0	41	27.3	99	33.6	0.02
Current vaginal symptoms	60	41.4	36	24.0	96	32.5	0.001
Current pain during VS	28	19.3	6	4.0	34	11.5	< 0.0001
Tested positive for an STI at baseline	41	28.3	15	10.0	56	19.0	< 0.0001
Gonorrhea	19	13.1	2	1.3	21	7.1	< 0.0001
Chlamydia	29	20.0	12	8.0	41	13.9	0.003
Syphilis (titer ≥1:8)	8	5.5	2	1.3	10	3.4	0.06

Numbers may not sum to column totals due to missing data; Percentages may not sum to 100 due to rounding or omission of one category for binary variables

*Abbreviations*: *STI* sexually transmitted infection, *USD* United States dollar, *VS* vaginal sex

### Vaginal washing practices

Overall, 74 and 39% of participants reported lifetime and past month vaginal washing, respectively (Table 2). While more participants in Tijuana (56%) reported past month vaginal washing compared to those in Ciudad Juarez (22%) (*p*-value< 0.0001), there was no difference in the frequency of vaginal washing by study site (*p*-value = 0.36), with 32% of participants overall reporting vaginal washing at least daily. Vaginal washing was most commonly practiced while bathing in both Tijuana (72%) and Ciudad Juarez (82%) (*p*-value = 0.34). However, more participants in Tijuana reported vaginal washing before (52% vs. 6%; *p*-value< 0.0001) or after (63% vs. 21%; *p*-value< 0.0001) vaginal sex. Of all participants who reported vaginal washing before vaginal sex in the past month, 80 and 76% reported washing before most or all vaginal sex acts with regular and non-regular clients, respectively. Of all participants who reported vaginal washing after vaginal sex in the past month, 75 and 74% reported washing after most or all vaginal sex acts with regular and non-regular clients, respectively.

**Table 2:** Vaginal washing practices among HIV-negative female sex workers in Tijuana and Ciudad Juarez, Mexico (*N* = 295)

	Tijuana		Ciudad Juarez		Total		*p*-value
	n	%	n	%	n	%	
Vaginal washing							
Lifetime	123	84.8	94	62.7	217	73.6	< 0.0001
Past month	81	55.9	33	22.0	114	38.6	< 0.0001
Frequency of vaginal washing in the past month^a^							0.36
Less than weekly	24	29.6	7	21.2	31	27.2	
Once a week	15	18.5	10	30.3	25	21.9	
Several times a week	14	17.3	8	24.2	22	19.3	
Once a day	13	16.1	2	6.1	15	13.2	
Several times a day	15	18.5	6	18.2	21	18.4	
Timing of vaginal washing in the past month^a^							
Before vaginal sex	42	51.9	2	6.1	44	38.6	< 0.0001
Washed before most or all (≥75%) of VS acts with regular clients	33	78.6	2	100.0	35	79.6	
Washed before most or all (≥75%) of VS acts with non-regular clients	27	75.0	2	100.0	29	76.3	
After vaginal sex	51	63.0	7	21.2	58	50.9	< 0.0001
Washed after most or all (≥75%) of VS acts with regular clients	39	79.6	3	42.9	42	75.0	
Washed after most or all (≥75%) of VS acts with non-regular clients	33	78.6	3	42.9	36	73.5	
Around menstruation	21	25.9	7	21.2	28	24.6	0.64
While bathing for personal hygiene	58	71.6	27	81.8	85	74.6	0.34
Solutions used for vaginal washing in the past month^a^							< 0.0001
Commercial solutions only	15	20.3	25	78.1	40	37.7	
Non-commercial solutions only	14	18.9	1	3.1	15	14.2	
Both commercial and non-commercial solutions	45	60.8	6	18.8	51	48.1	

*Abbreviations*: *VS* vaginal sex

^a^ Among participants who reported vaginal washing in the past month

More participants in Ciudad Juarez (78%) reported using only commercial solutions for vaginal washing in the past month, while more participants in Tijuana reported using both commercial and non-commercial solutions (61%) (*p*-value< 0.0001). Participants in each site reported using a variety of commercial solutions for vaginal washing. Intimo Shampoo Reafirmante (26%) was the most commonly used commercial solution in Ciudad Juarez followed by Intimo Jabon Fresco (23%), while Summer’s Eve (50%) was the most commonly used commercial solution in Tijuana followed by Benzal Odor-Block Spray Desodorante (18%). Commercial solution application methods also varied by study site. Seventy-five percent of participants who reported vaginal washing with commercial solutions in the past month in Tijuana most often reported using vaginal douche kits or devices that came with the commercial solution used (vs. 42% in Ciudad Juarez; *p*-value = 0.002), while 61% of participants who reported vaginal washing with commercial solutions in the past month in Ciudad Juarez most often reported using reusable vaginal douche kits or devices that did not come with the commercial solution used (vs. 3% in Tijuana; *p*-value< 0.0001). Water and soap (59%) was the most commonly used non-commercial solution among participants in Tijuana followed by vinegar (37%) and water and herbs (22%), while vinegar (29%) and lemon juice (29%) were the most commonly used non-commercial solutions among participants in Ciudad Juarez. A large proportion of participants who reported vaginal washing in the past month with non-commercial solutions in both Tijuana (66%) and Ciudad Juarez (43%) reported using their fingers to apply the non-commercial solutions used (*p*-value = 0.25); however, 57% of participants in Ciudad Juarez reported using reusable vaginal douche kits or devices to apply the non-commercial solutions used compared to only 15% of participants in Tijuana (*p*-value = 0.02).

Participants reported a variety of motivators and barriers to vaginal washing in the past month. Among those who reported vaginal washing in the past month (Additional file 1), the most common reason for performing this practice in both Tijuana (91%) and Ciudad Juarez (85%) was to *“promote personal hygiene and feel clean or fresh”* (*p*-value = 0.32) followed by to “*eliminate or reduce odor”* in Tijuana (42% vs. 9%; *p*-value = 0.001) and to “*prevent infection”* in Ciudad Juarez (55% vs. 31%; *p*-value = 0.02). More participants in Tijuana also reported vaginal washing to “*clean up sweat, vaginal fluids, or semen after vaginal sex”* (31% vs. 6%; *p*-value = 0.004) and to *“prepare for vaginal sex”* (17% vs. 0%; *p*-value = 0.01). Among those who did not report vaginal washing in the past month (Additional file 2), more participants in Ciudad Juarez *“did not know that women wash inside their vagina”* (31% vs. 16%; *p*-value = 0.03). Common reasons for not performing vaginal washing among participants in both Tijuana and Ciudad Juarez were *“vaginal washing is unnecessary”* (22 and 27% respectively; *p*-value = 0.45) and *“I do not like washing inside my vagina”* (16 and 15%, respectively; *p*-value = 0.81).

### Vaginal lubrication practices

Overall, 70 and 54% of participants reported lifetime and past month vaginal lubrication, respectively (Table 3). More participants in Tijuana (64%) than in Ciudad Juarez (45%) reported vaginal lubrication before or during sex in the past month (*p*-value = 0.001). Of those who reported vaginal lubrication in the past month, those in Tijuana did so with greater frequency, with 56% of participants in Tijuana reporting at least daily vaginal lubrication compared to 33% of participants in Ciudad Juarez (*p*-value = 0.004). The frequency of vaginal lubrication before or during sex with clients was also higher among participants in Tijuana, with 63 and 63% of participants in Tijuana reporting vaginal lubricant use before or during most or all vaginal sex acts with regular and non-regular clients, respectively, compared to only 25 and 20% of participants in Ciudad Juarez (*p*-values< 0.0001). Vaginal lubricant suppliers (*p*-value = 0.09) and appliers (*p*-value = 0.06) did not differ by study site, with 70% of participants overall reporting that they were the sole suppliers of the lubricants used in the past month and 71% of participants overall reporting that they were the sole appliers of the lubricants used in the past month. Although vaginal lubrication was defined for participants as pushing or placing products, such as creams, oils, or sexual lubricants, inside the vagina, when asked about the sites of vaginal lubricant application before or during vaginal sex in the past month, 50% of participants overall reported that vaginal lubricants were applied at multiple locations (58% outside the condom; 49% around the vagina; 23% inside the vagina). Thirty-seven percent of past month vaginal lubricant users in Ciudad Juarez reported that lubricant use rarely or sometimes interrupted sex compared to only 6% of those in Tijuana (*p*-value< 0.0001); however, less than 5% reported that lubricant use interrupted sex at least 50% of the time.

**Table 3:** Vaginal lubrication practices among HIV-negative female sex workers in Tijuana and Ciudad Juarez, Mexico (*N* = 295)

	Tijuana		Ciudad Juarez		Total		*p*-value
	n	%	n	%	n	%	
Vaginal lubrication before or during VS							
Lifetime	116	80.0	90	60.0	206	69.8	0.0002
Past month	92	63.5	67	44.7	159	53.9	0.001
Frequency of lubricant use before or during VS in the past month^a^							0.004
Less than weekly	9	9.8	6	9.0	15	9.4	
Once a week	6	6.5	6	9.0	12	7.6	
Several times a week	26	28.3	33	49.3	59	37.1	
Once a day	3	3.3	6	9.0	9	5.7	
Several times a day	48	52.2	16	23.9	64	40.3	
Vaginal lubricant use with clients in the past month^a^	89	98.9	63	96.9	152	98.1	0.57
Used vaginal lubricants before or during most or all (≥75%) VS acts with regular clients	54	62.8	16	25.4	70	47.0	< 0.0001
Used vaginal lubricants before or during most or all (≥75%) VS acts with non-regular clients	52	63.4	13	20.0	65	44.2	< 0.0001
Who supplied lubricants used before or during VS in the past month?^a^							0.09
Participant	71	77.2	41	61.2	112	70.4	
Clients or other sex partners	11	12.0	15	22.4	26	16.4	
Both the participant and her clients or other sex partner	10	10.9	11	16.4	21	13.2	
Who applied lubricants used before or during VS in the past month?^a^							0.06
Participant	72	78.3	41	61.2	113	71.1	
Clients or other sex partners	7	7.6	10	14.9	17	10.7	
Both the participant and her clients or other sex partner	13	14.1	16	23.9	29	18.2	
Where was lubricant applied before or during VS in the past month?^a^							
Around my vagina	40	43.5	38	56.7	78	49.1	0.10
Inside my vagina	25	27.2	12	17.9	37	23.3	0.17
Directly on my client or sex partners penis	7	7.6	22	32.8	29	18.2	< 0.0001
On the outside of the condom	64	69.6	28	41.8	92	57.9	0.001
Inside the condom	11	12.0	6	9.0	17	10.7	0.61
Applied lubricant at multiple locations	47	51.1	32	47.8	79	49.7	0.68
Lubricant use before or during VS interrupted sex in the past month^a^							< 0.0001
Never	82	89.1	41	61.2	123	77.4	
Rarely (1–24% of the time)	2	2.2	10	14.9	12	7.6	
Sometimes (25–49% of the time)	3	3.3	15	22.4	18	11.3	
Usually (50–74% of the time)	1	1.1	0	0.0	1	0.6	
Most of the time (75–99% of the time)	3	3.3	1	1.5	4	2.5	
Always (100% of the time)	1	1.1	0	0.0	1	0.6	
Type of lubricant used before or during VS in the past month^a^							
Water-based lubricants	62	68.1	39	58.2	101	63.9	0.20
Silicone-based lubricants	11	12.1	7	10.5	18	11.4	0.81
Oil-based lubricants	22	24.2	31	46.3	53	33.5	0.004
Lubricant used before or during VS in the past month^a^							0.05
Commercial lubricants only	57	63.3	31	48.4	88	57.1	
Non-commercial lubricants only	2	2.2	0	0.0	2	1.3	
Both commercial and non-commercial lubricants	31	34.4	33	51.6	64	41.6	

*Abbreviations*: *VS* vaginal sex

^a^Among participants who reported vaginal lubrication in the past month

More participants (63%) in Tijuana reported using only commercial products for vaginal lubrication in the past month, while 48 and 52% of participants in Ciudad Juarez reported using only commercial products and both commercial and non-commercial products, respectively (*p*-value = 0.05). Participants in each site reported using a variety of commercial products for vaginal lubrication. Sico Soft Lube Original (26%) was the most commonly used commercial product for vaginal lubrication in Tijuana followed by Equate Personal Lubricant Jelly (20%) and Lubifem Lubricante Vaginal (17%), while KY Jelly (17%) was the most commonly used commercial product for vaginal lubrication in Ciudad Juarez followed by Multi-O-Gel (16%), Prudence Lub Naranja (13%), and Benzal Gel Lubricante Vaginal Original (13%). Non-commercial products used for vaginal lubrication in the past month did not differ by study site. Overall, oils (e.g., cooking oil, baby oil) were the most commonly used (53%) non-commercial lubricants followed by petroleum jelly (39%) and creams or lotions (27%). All participants in both study sites reported applying both commercial and non-commercial vaginal lubricants using their fingers.

Among those who reported vaginal lubrication in the past month (Additional file 3), common reasons for performing this practice in both Tijuana and Ciudad Juarez were to *“reduce vaginal dryness”* (83 and 76%, respectively; *p*-value = 0.32) and to *“reduce discomfort or pain during vaginal sex”* (41 and 54%; respectively; *p*-value = 0.12). However, more participants in Ciudad Juarez than in Tijuana also reported performing this practice because a *“client or sex partner requested it in preparation for vaginal sex”* (52% vs. 14%; *p*-value<0.0001), a *“client or sex partner requested it to enhance their sexual pleasure”* (48% vs. 9%; *p*-value<0.0001), to *“prepare for vaginal sex”* (40% vs. 14%; *p*-value = 0.0002), and to *“enhance my sexual pleasure”* (28% vs. 9%; *p*-value = 0.002). Among participants who did not perform vaginal lubrication in the past month (Additional file 4), common reasons for not doing so in both Tijuana and Ciudad Juarez were *“vaginal lubrication is unnecessary”* (36 and 28%, respectively; *p*-value = 0.35) and *“I do not like using vaginal lubricants”* (30 and 26%, respectively; *p*-value = 0.64). Other reasons for not performing this practice that were more commonly reported in Ciudad Juarez compared to Tijuana were *“I use lubricated condoms”* (82% vs. 15%; *p*-value< 0.0001) and *“my clients or other sex partners prefer dry sex and dislike it when I use vaginal lubricants”* (22% vs. 4%; *p*-value = 0.01).

### Overlapping vaginal practices

Overall, 35% of participants reported no vaginal washing and no vaginal lubrication in the past month, 12% reported vaginal washing only, 27% reported vaginal lubrication only, and 27% reported both vaginal washing and vaginal lubrication. More participants in Tijuana (42%) reported performing both vaginal washing and vaginal lubrication in the past month than reported performing both in Ciudad Juarez (12%; *p*-value< 0.0001).

### Predictors of vaginal washing and vaginal lubrication

Potential predictors of vaginal washing and lubrication identified in bivariate analyses are presented in Table 4. In multivariable analyses examining the effect of potential predictors on vaginal washing in the past month (Table 5), vaginal washing was positively associated with being from Tijuana (adjusted odds ratio [AOR] = 4.35, 95% confidence interval [CI]: 2.60, 7.30), older age (AOR = 1.04 per year, 95% CI: 1.01, 1.06), and vaginal lubrication in the past month (AOR = 2.99, 95% CI: 1.67, 5.35), while vaginal washing was negatively associated with being born in the same state as the study site (AOR = 0.50, 95% CI: 0.31, 0.82), reporting an average monthly income ≥3500 pesos (AOR = 0.53, 95% CI: 0.28, 1.00) and hazardous alcohol consumption (AOR = 0.56, 95% CI: 0.33, 0.95). In multivariable analyses examining the effect of potential predictors on vaginal lubrication in the past month (Table 5), vaginal lubrication was positively associated with being from Tijuana (AOR = 2.21, 95% CI: 1.37, 3.54) and vaginal washing in the past month (AOR = 2.91, 95% CI: 1.64, 5.18), while vaginal lubrication was negatively associated with being born in the same state as the study site (AOR = 0.47, 95% CI: 0.29, 0.75).

**Table 4:** Unadjusted associations between the predictors of interest and vaginal washing and lubrication in the past month among female sex workers in Tijuana and Ciudad Juarez, Mexico

	Vaginal washing		Vaginal lubrication	
	OR	95% CI	OR	95% CI
Sociodemographics				
Age (years)	1.04	1.01, 1.06	1.01	0.99, 1.04
Tijuana study site	4.49	2.70, 7.45	2.15	1.35, 3.43
Born in same state as study site	0.47	0.29, 0.76	0.47	0.29, 0.74
Completed secondary school	0.74	0.46, 1.19	0.89	0.56, 1.41
Married/common-law relationship	0.89	0.53, 1.52	0.96	0.57, 1.61
Number of children	1.12	0.99, 1.28	1.00	0.88, 1.13
Average monthly income ≥3500 pesos (~ 172 USD)	0.25	0.15, 0.42	0.67	0.41, 1.12
Substance Use				
Illicit drug use (past month)	1.25	0.77, 2.03	0.89	0.55, 1.42
Any hazardous alcohol consumption (past 12 months)	0.51	0.31, 0.82	0.67	0.42, 1.06
Sex work characteristics				
Main sex work location identified as the street	3.70	2.26, 6.07	1.69	1.06, 2.71
Difference in USD earned for condomless VS	1.02	1.00, 1.04	1.00	0.98, 1.02
Ever forced to have sex or physically abused by a client	0.98	0.60, 1.59	0.96	0.60, 1.55
Number of clients (past month)	1.00	0.99, 1.01	1.00	0.99, 1.01
Any drug/alcohol use during sex with clients (past month)	0.79	0.49, 1.28	0.51	0.31, 0.82
% of VS acts with clients with condoms (past month)	2.43	1.15, 5.16	1.79	0.87, 3.66
Reproductive/sexual health				
Routine gynecological exam (past 6 months)	0.77	0.37, 1.61	0.84	0.42, 1.68
Contraceptive use other than condoms (past 6 months)	0.41	0.25, 0.66	0.53	0.33, 0.84
HIV/STI testing (past 6 months)	1.74	1.06, 2.85	1.71	1.04, 2.80
Current vaginal symptoms	0.71	0.43, 1.19	1.48	0.90, 2.43
Current pain during VS	1.69	0.83, 3.47	2.24	1.03, 4.87
Tested positive for an STI	1.03	0.57, 1.88	0.76	0.42, 1.35
Vaginal practices (past month)				
Vaginal lubrication	3.04	1.85, 5.00	–	–
Vaginal washing	–	–	3.04	1.85, 5.00

*Abbreviations*: *CI* confidence interval, *OR* odds ratio, *STI* sexually transmitted infection, USD United States dollar, *VS* vaginal sex

**Table 5:** Adjusted associations between the predictors of interest and vaginal washing and lubrication in the past month among female sex workers in Tijuana and Ciudad Juarez, Mexico

	Vaginal washing		Vaginal lubrication	
	AOR	95% CI	AOR	95% CI
Sociodemographics				
Age (years)^a^	1.04	1.01, 1.06		
Tijuana study site^a^	4.35	2.60, 7.30	2.21	1.37, 3.54
Born in same state as study site^a^	0.50	0.31, 0.82	0.47	0.29, 0.75
Average monthly income ≥3500 pesos (~ 172 USD)^b^	0.53	0.28, 1.00		
Substance Use				
Any hazardous alcohol consumption (past 12 months)^b^	0.56	0.33, 0.95		
Sex work characteristics				
Main sex work location identified as the street^c^	0.84	0.36, 1.97	0.65	0.28, 1.52
Difference in USD earned for condomless VS^c^	1.02	0.99, 1.05		
Any drug/alcohol use during sex with clients (past month)^c^			0.70	0.38, 1.28
% of VS acts with clients with condoms (past month)^c^	1.64	0.68, 3.97		
Reproductive/sexual health				
Contraceptive use other than condoms (past 6 months)^d^	1.39	0.67, 2.86	0.74	0.39, 1.40
HIV/STI testing (past 6 months)^d^	1.51	0.85, 2.70	1.46	0.86, 2.48
Current pain during VS^d^			1.72	0.74, 3.98
Vaginal practices (past month)				
Vaginal lubrication^e^	2.99	1.67, 5.35	–	–
Vaginal washing^e^	–	–	2.91	1.64, 5.18

*Abbreviations*: *AOR* adjusted odds ratio, *CI* confidence interval, *STI* sexually transmitted infection, *USD* United States dollar, *VS* vaginal sex

^a^Adjusted for intervention group assignment and age

^b^ Adjusted for variables included in a; additionally adjusted for study site, birthplace, education, marital status, number of children, and income

^c^ Adjusted for variables included in a and b; additionally adjusted for illicit drug use (past month) and hazardous alcohol consumption (past 12 months)

^d^ Adjusted for variables included in a, b, and c; additionally adjusted for number of clients (past month) and percentage of VS acts with clients with condoms (past month)

^e^ Adjusted for variables included in a, b, c, and d; additionally adjusted for HIV/STI testing (past 6 months) and STI status at baseline

## Discussion

We examined vaginal washing and vaginal lubrication among FSWs in Tijuana and Ciudad Juarez, Mexico. Overall, we found a moderate (39%) and high (54%) prevalence of vaginal washing and vaginal lubrication in the past month, respectively, suggesting that vaginal PrEP products formulated as douches or gels may be acceptable HIV prevention methods to many FSWs in the Mexico-US border region. We also identified several predictors of each vaginal practice, which provide insight on the characteristics of FSWs for whom these vaginal PrEP products may be most acceptable. Our findings related to practice-specific behaviors also highlight important considerations for the development and implementation of vaginal PrEP products that can be readily integrated into FSWs’ existing vaginal practices.

We found a strong association between vaginal washing and vaginal lubrication. Although we did not specifically collect information on vaginal washing before or after sexual encounters during which vaginal lubricants were used, the frequency with which each practice occurred in the context of sex with regular and non-regular clients, particularly in Tijuana, suggests that there may have been considerable overlap in their performance in the context of a single sexual encounter. This finding highlights the need to consider existing vaginal practices to ensure the development of safe and effective vaginal PrEP products [23]. If vaginal washing or vaginal lubrication continue to be performed in conjunction with the use of efficacious vaginal gel- or douche-based PrEP products, the solutions or products used for these vaginal practices could interact with or dilute the effectiveness of vaginal PrEP products [23]. Given that vaginal washing is linked to BV and recent evidence suggests that BV-associated bacteria reduce the efficacy of vaginal PrEP formulated as tenofovir-containing gels [18], alternative ARV formulations for vaginal gel-based PrEP products may be more effective for FSWs who practice vaginal washing. However, because vaginal washing has also been linked to HIV infection [39], and is hypothesized to increase women’s vulnerability to HIV by drying out the vagina and altering the vaginal microbiome or disrupting the vaginal epithelium [56], these vaginal practices could undermine the protective benefits conferred by efficacious vaginal PrEP products regardless of their ARV formulation. Furthermore, some participants reported using oil-based lubricants, which can damage latex condoms and facilitate HIV transmission. Therefore, it is critical that continued development of vaginal PrEP products considers FSWs’ current vaginal washing and lubrication practices to ensure that they meet FSWs’ vaginal needs such that FSWs are not motivated to apply additional solutions or products vaginally [23]. Due to the timing of HIV/STI testing and assessment of vaginal symptoms and pain during vaginal sex (i.e., baseline) relative to vaginal washing and lubrication practices (i.e., approximately 1 month after baseline), we could not examine the impact of these vaginal practices on reproductive and sexual health. However, given the frequency of these practices and the range of commercial and non-commercial solutions and products FSWs in our sample reported using for vaginal washing and lubrication, it is imperative that future research investigates the safety of these solutions and products, including their impact on gynecologic and reproductive health, HIV/STI risk, and vaginal PrEP product efficacy. This work could help inform interventions to reduce the risk of HIV/STIs in the context of vaginal washing and lubrication practices as well as messages on the appropriate use of vaginal PrEP products and the potential risks associated with the concurrent use of other vaginal solutions and products.

We also identified variation across study sites with respect to vaginal practice frequency, timing, and motivations. Vaginal washing in the past month was more common in Tijuana, where 56% of participants reported vaginal washing compared to only one in five participants in Ciudad Juarez. Most participants in Tijuana and Ciudad Juarez who performed vaginal washing in the past month reported doing so to promote their own personal hygiene. However, over half of Tijuana participants who performed vaginal washing in the past month did so in the context of sex. Differences in the timing of vaginal washing between Tijuana and Ciudad Juarez may be attributable to regional variation in norms surrounding the practice, as has been described in previous research [30, 31]. Vaginal lubrication before or during sex in the past month, on the other hand, was common in both Ciudad Juarez (45%) and Tijuana (64%). In both cities, vaginal lubrication was most commonly performed to reduce vaginal dryness; however, over half of participants in Tijuana who performed vaginal lubrication in the past month did so at least daily compared to only one third of participants in Ciudad Juarez. Prior work suggests that expectations related to the degree of vaginal lubrication during sex vary across regions [32], which may explain the variation with respect to the frequency of vaginal lubrication observed across study sites within our sample. Taken together, these findings suggest that using vaginal douche-based PrEP products before and after sex may be easily integrated into the existing vaginal practices of FSWs like those enrolled in Tijuana, while vaginal PrEP formulated as a lubricating gel for use before and after sex may be acceptable to many FSWs in Tijuana and Ciudad Juarez. Nevertheless, the varying vaginal practices across study sites should be considered in the continued development of vaginal PrEP products to ensure that FSWs have options and can choose the product they find most acceptable or congruent with their existing behaviors.

Consistent with prior research conducted in the United States, we found that vaginal washing was associated with older age [57–59] and earning a lower income [59–61]. FSWs who reported hazardous alcohol consumption were also less likely to perform vaginal washing, suggesting that uptake and adherence to vaginal douche-based PrEP products may be more challenging for substance-involved FSWs. Given that such FSWs are often most socially marginalized and vulnerable to HIV infection [2], interventions to support their uptake of PrEP products or alternative prevention strategies may be needed.

Given that a number of structural factors often limit FSWs’ ability to negotiate condom use with clients [2], one attractive feature of vaginal PrEP products is their potential to be controlled by the user. Less than 5% of participants in either study site reported that their vaginal washing in the past month was motivated by requests from clients or other sex partners. Although requests from clients or other sex partners were commonly reported as motivators of vaginal lubrication in the past month by participants in Ciudad Juarez, most participants in both cities who reported vaginal lubrication in the past month did not solely rely on their clients or sex partners to supply or apply the vaginal lubricants used. As such, vaginal gel- and douche-based PrEP products may be feasibly incorporated into FSWs’ existing vaginal practices as user-controlled HIV prevention methods.

Furthermore, the fact that less than 20 and 5% of participants overall reported past month use of only non-commercial solutions for vaginal washing and vaginal lubrication, respectively, suggests that many FSWs in the border region who perform these practices are accustomed to doing so with commercial products. Prior research conducted with FSWs in India and Kenya participating in oral PrEP feasibility studies or clinical trials suggests that barriers to PrEP uptake and adherence may stem from the social and economic consequences related to being perceived as HIV-positive by friends, family, and clients or other sex partners if they are seen taking oral PrEP [62, 63]. By incorporating HIV prevention methods into existing vaginal practices, such barriers may be mitigated among FSWs in the Mexico-US border region, particularly if vaginal gel- and douche-based PrEP products are packaged and priced similarly to currently used commercial vaginal products, and can be purchased from vendors (e.g., corner stores or pharmacies) that sell these products to FSWs in the region.

To ensure that a sufficient amount of PrEP is delivered inside the vagina, clinical trials have primarily evaluated vaginal gel-based PrEP delivered using applicators [64, 65], and it is likely that douching devices would need to be used to deliver a sufficient amount of vaginal douche-based PrEP inside the vagina. In our study, most participants applied the commercial solutions used for vaginal washing using disposable or reusable douche kits or devices designed to introduce solution into the vagina. This practice suggests that vaginal douche-based PrEP delivery vehicles may be familiar to most FSWs who use commercial solutions to perform vaginal washing. However, many participants used their fingers to apply non-commercial solutions for vaginal washing and nearly all participants used their fingers to apply commercial and non-commercial products for vaginal lubrication. Findings from previous vaginal gel-based PrEP acceptability research suggest that ease of use, portability, storage requirements, availability of water to clean reusable delivery vehicles, the potential for discreet disposal of single-use delivery vehicles, and the cost associated with different delivery vehicles (e.g., disposable vs. reusable) will likely influence the acceptability of vaginal PrEP products [64, 66, 67]. These factors may similarly affect uptake of vaginal gel- or douche-based PrEP products among FSWs in the Mexico-US border region, particularly those accustomed to using their fingers for vaginal washing and lubrication. As such, FSWs may need access to a range of delivery vehicles for each vaginal PrEP product formulation so that individual FSWs can choose the formulation and delivery vehicle combination they find most acceptable and easy to use.

It is important to note that our results differ in several ways from those reported by a previous study that examined intravaginal practices among FSWs in Tijuana and Ciudad Juarez [28]. In contrast to FSWs included in our study, in the previous study, a greater proportion of FSWs in Ciudad Juarez (53%) than in Tijuana (33%) reported performing intravaginal practices in the past 6 months [28]. Furthermore, while data on the use of commercial solutions were not reported by the previous study, unlike our findings with respect to the use of non-commercial solutions for vaginal washing, the previous study found that use of homemade solutions for intravaginal practices was higher in Ciudad Juarez (79%) than in Tijuana (30%) [28]. While these discrepancies may reflect variation in norms affecting FSWs’ vaginal washing and lubrication practices both across and within settings over time, they may also be explained by a number of important differences between the present and previous studies. First, our sample was slightly older and on average reported a higher monthly income, and was not restricted to FSWs who inject drugs. Second, the previous study did not distinguish between vaginal washing and lubrication and only measured intravaginal practices, which were defined as the “insertion of liquid, suppositories, or other materials into the vagina for any reason.” This may have masked important differences in the prevalence of each vaginal practice and behaviors associated with the performance of each practice. Third, unlike the previous study, we presented participants with an exhaustive list that included pictures of commercially available solutions and products for vaginal washing and lubrication, which may have improved recall of their use in our study relative to the previous study.

However, our study also has several limitations. First, due to limited computer literacy within our sample, surveys were interviewer-administered via CAPI instead of audio-computer-assisted self-interview (ACASI). Although interviewers received training on how to build rapport with participants and encourage open communication, social desirability bias and recall bias may have led to under-reporting of vaginal practices and sexual risk behaviors. Second, although vaginal washing and lubrication were not specifically discussed during the interactive sexual risk reduction counseling session conducted at baseline, the session could have affected participants’ vaginal practices in the following month by raising their awareness about sexual health issues. Third, although we collected detailed practice-specific data related to vaginal washing and lubrication, to limit the burden on participants, we did not collect information on the frequency with which each reported commercial and non-commercial solution and product was used or the frequency with which different application methods were employed. Fourth, although open ended questions were used to collect information on motivators and barriers to vaginal washing and lubrication that were not directly assessed in the survey, in-depth qualitative interviews might have provided a more comprehensive understanding of these issues and their potential impact on vaginal PrEP uptake. Finally, because anal sex is highly stigmatized in this setting, 54% of participants refused to answer questions about anal sex. As such, we were unable to estimate the prevalence of FSWs in the Mexico-US border region who engage in anal sex. The extent to which vaginal PrEP products will provide sufficient protection or whether rectal or multi-compartment (i.e., vagina and rectum) or systemic PrEP products are needed within this population remains unknown and merits further research.

## Conclusions

Despite these limitations, the collection of detailed data on vaginal washing and lubrication practices from high-risk FSWs in two northern Mexico border cities are among our studies many strengths. Our findings suggest that it may be possible to leverage FSWs existing vaginal washing and lubrication practices to deliver PrEP vaginally in the form of a douche or gel within this vulnerable population. However, FSWs’ practice-specific behaviors must be considered in the development and implementation of acceptable, safe, and effective vaginal PrEP products to ensure that uptake and adherence are sufficient to provide individual-level protection and population-level reductions in HIV incidence.

## Additional files

Additional file 1: Reasons for performing vaginal washing in the past month among HIV-negative female sex works in Tijuana (TJ) and Ciudad Juarez (CJ), Mexico (*N* = 114). * *p*-value < 0.05. (PDF 130 kb)

Additional file 2: Reasons for not performing vaginal washing in the past month among HIV-negative female sex works in Tijuana (TJ) and Ciudad Juarez (CJ), Mexico (*N* = 181). Other: I did not have time (3% TJ; 2% CJ), I was too lazy (0% TJ; 6% CJ), I forgot or was concerned about other things (8% TJ; 15% CJ), washing causes vaginal dryness and discomfort (6% TJ; 1% CJ), I cannot afford the solutions needed for vaginal washing (8% TJ; 0% CJ), I am too scared to wash inside my vagina (2% TJ; 1% CJ), I do not know how to wash inside my vagina (0% TJ; 1% CJ), I did not have any vaginal infections or symptoms (0% TJ; 7% CJ). * *p*-value < 0.05. (PDF 34 kb)

Additional file 3: Reasons for performing vaginal lubrication in the past month among HIV-negative female sex works in Tijuana (TJ) and Ciudad Juarez (CJ), Mexico (*N* = 159). Other: prevent condom breakage (10% TJ; 5% CJ). * *p*-value < 0.05. (PDF 120 kb)

Additional file 4: Reasons for not performing vaginal lubrication in the past month among HIV-negative female sex works in Tijuana (TJ) and Ciudad Juarez (CJ), Mexico (*N* = 136). Other: Lubricants irritate my vagina (8% TJ; 1% CJ), I did not have time (2% TJ; 0% CJ), I cannot afford vaginal lubricants (2% TJ; 0% CJ), I did not have any vaginal dryness (2% TJ; 0% CJ), vaginal lubricants cause condoms to slip off (2% TJ; 0% CJ), I do not know how to use vaginal lubricants (0% TJ; 1% CJ), my clients did not bring vaginal lubricants (0% TJ; 2% CJ). * *p*-value < 0.05. (PDF 34 kb)

## Abbreviations

AOR: Adjusted odds ratio
ARV: Antiretroviral
BV: Bacterial vaginosis
CAPI: Computer-assisted personal interviewing
CI: Confidence interval
FSW: Female sex worker
IQR: Interquartile range
OR: Odds ratio
PrEP: Pre-exposure prophylaxis
RPR: Rapid plasma regain
STI: Sexually transmitted infection
TP: Treponemal
US: United States
