# Supplementary figures and images for: Vaginal washing and lubrication among female sex workers in the Mexico-US border region: implications for the development of vaginal PrEP for HIV prevention
Source: BMC Public Health. 2018 Aug 14;18:1009. doi: 10.1186/s12889-018-5946-z (PMC6092873; doi:10.1186/s12889-018-5946-z)

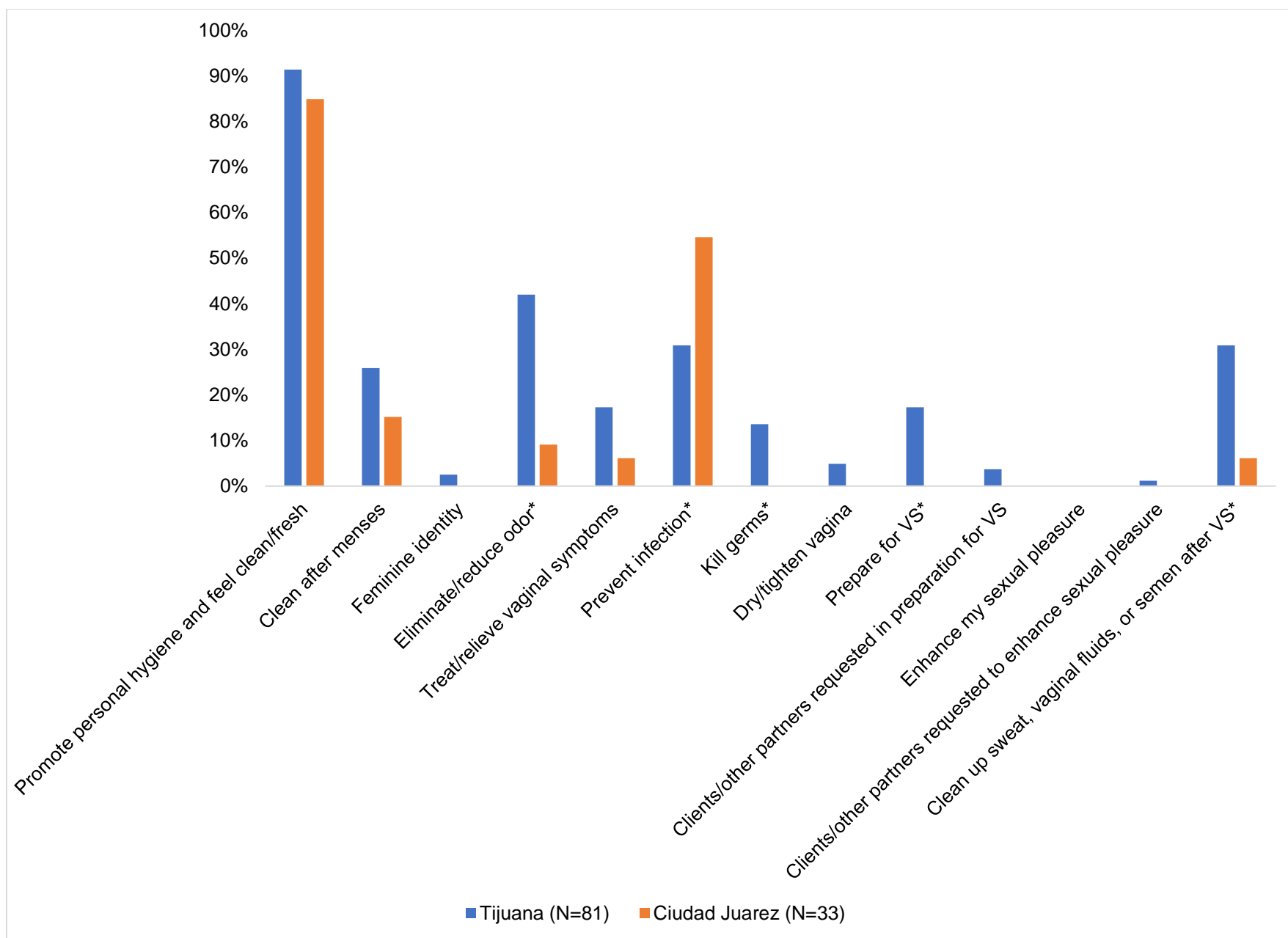

Supplement: Supplementary file 1 — Reasons for performing vaginal washing in the past month among HIV-negative female sex works in Tijuana (TJ) and Ciudad Juarez (CJ), Mexico (N = 114). * p-value < 0.05. (PDF 130 kb) [file 12889_2018_5946_MOESM1_ESM.pdf]

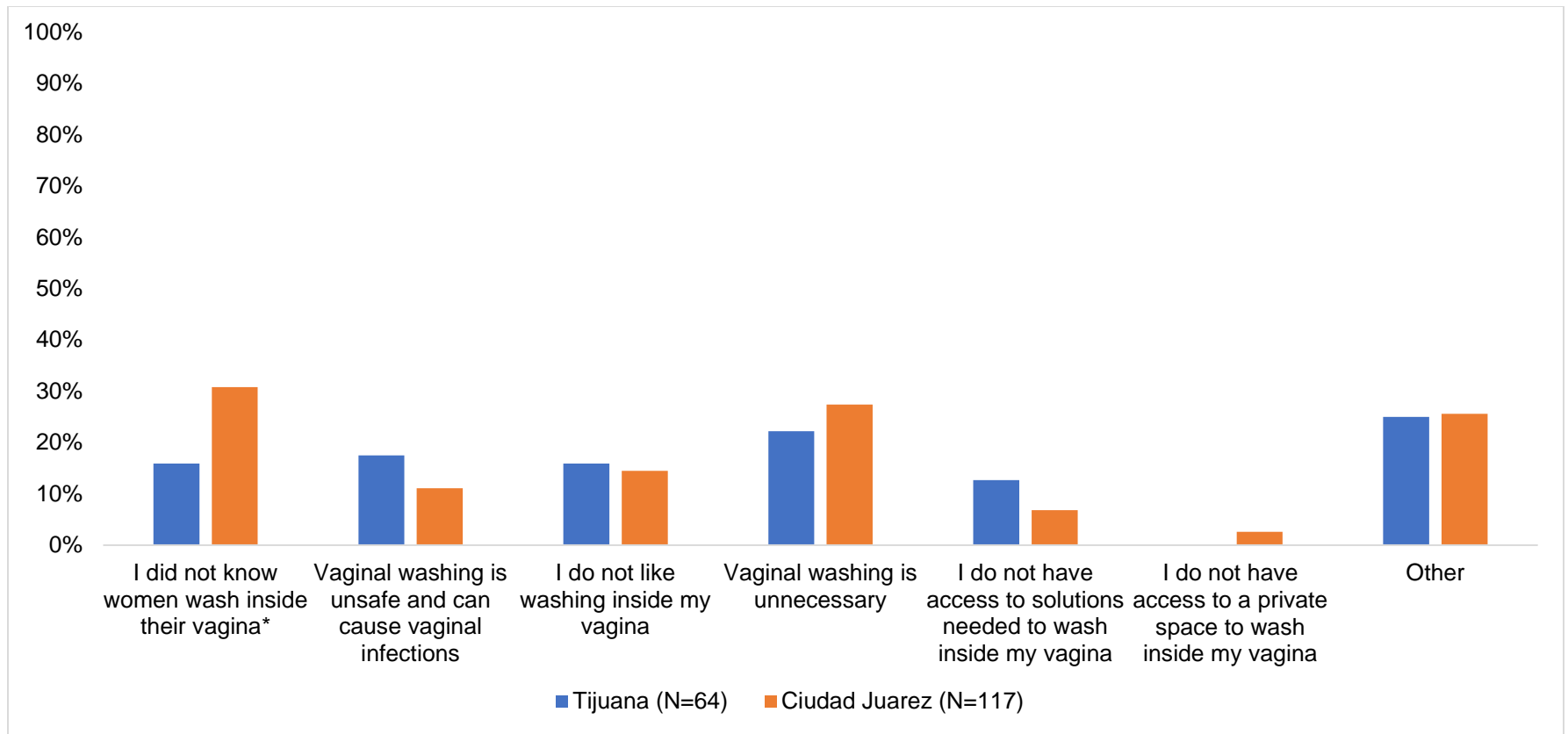

Supplement: Supplementary file 2 — Reasons for not performing vaginal washing in the past month among HIV-negative female sex works in Tijuana (TJ) and Ciudad Juarez (CJ), Mexico (N = 181). Other: I did not have time (3% TJ; 2% CJ), I was too lazy (0% TJ; 6% CJ), I forgot or was concerned about other things (8% TJ; 15% CJ), washing causes vaginal dryness and discomfort (6% TJ; 1% CJ), I cannot afford the solutions needed for vaginal washing (8% TJ; 0% CJ), I am too scared to wash inside my vagina (2% TJ; 1% CJ), I do not know how to wash inside my vagina (0% TJ; 1% CJ), I did not have any vaginal infections or symptoms (0% TJ; 7% CJ). * p-value < 0.05. (PDF 34 kb) [file 12889_2018_5946_MOESM2_ESM.pdf]

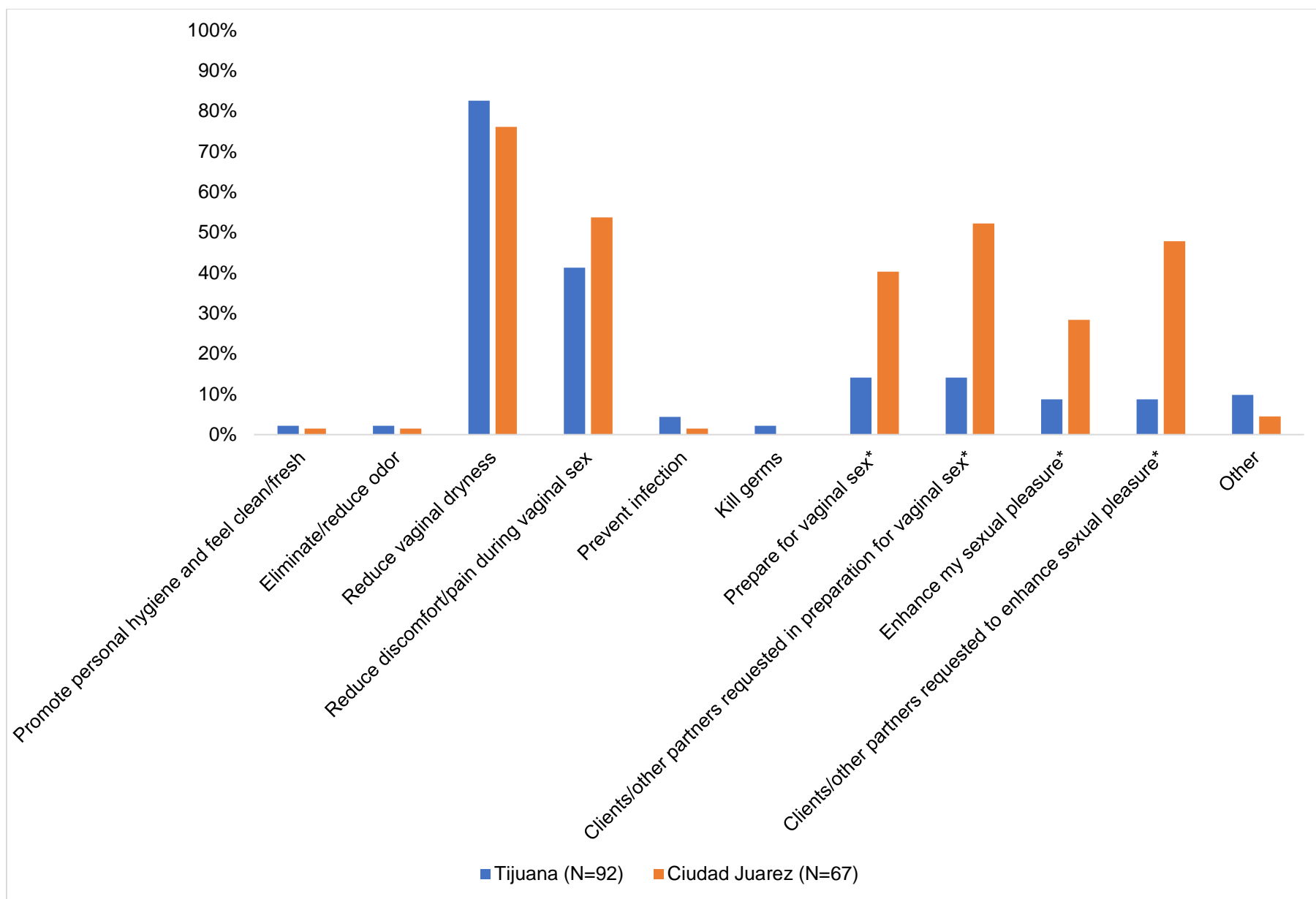

Supplement: Supplementary file 3 — Reasons for performing vaginal lubrication in the past month among HIV-negative female sex works in Tijuana (TJ) and Ciudad Juarez (CJ), Mexico (N = 159). Other: prevent condom breakage (10% TJ; 5% CJ). * p-value < 0.05. (PDF 120 kb) [file 12889_2018_5946_MOESM3_ESM.pdf]

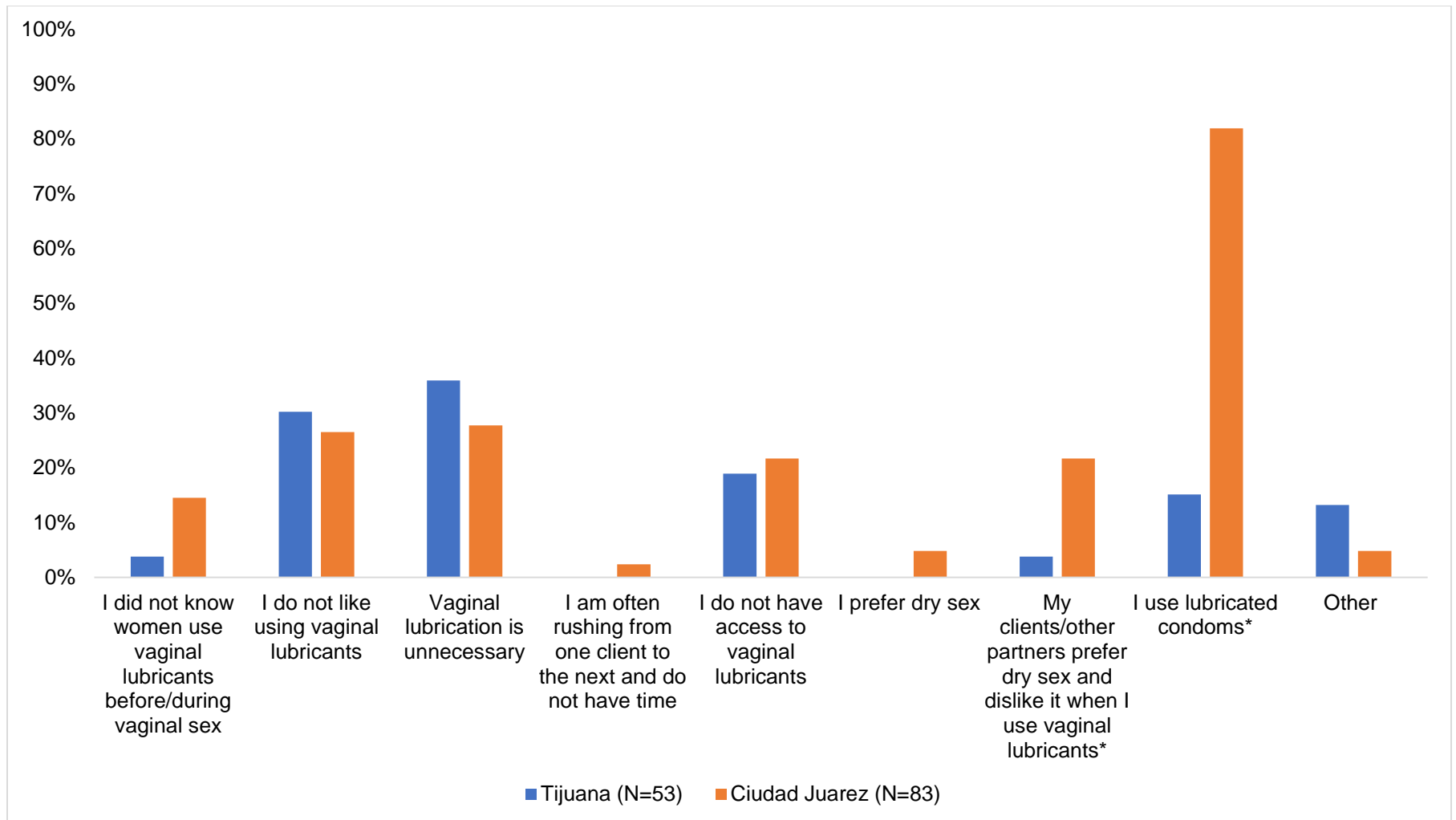

Supplement: Supplementary file 4 — Reasons for not performing vaginal lubrication in the past month among HIV-negative female sex works in Tijuana (TJ) and Ciudad Juarez (CJ), Mexico (N = 136). Other: Lubricants irritate my vagina (8% TJ; 1% CJ), I did not have time (2% TJ; 0% CJ), I cannot afford vaginal lubricants (2% TJ; 0% CJ), I did not have any vaginal dryness (2% TJ; 0% CJ), vaginal lubricants cause condoms to slip off (2% TJ; 0% CJ), I do not know how to use vaginal lubricants (0% TJ; 1% CJ), my clients did not bring vaginal lubricants (0% TJ; 2% CJ). * p-value < 0.05. (PDF 34 kb) [file 12889_2018_5946_MOESM4_ESM.pdf]
